# Use of copper intrauterine devices in the immediate postpartum period: a prospective longitudinal study

**DOI:** 10.1590/0034-7167-2025-0153

**Published:** 2026-07-06

**Authors:** Christiane Borges do Nascimento Chofakian, Giovana Nonato, Raphaela Romão Teixeira de Souza, Rosemeire Sartori de Albuquerque, Alícia Rafaelly Vilefort Sales, Ana Luiza Vilela Borges

**Affiliations:** I Universidade de São Paulo. São Paulo, São Paulo, Brazil

**Keywords:** Intrauterine Devices, Family Planning Policy, Contraception, Sex Education, Postpartum Period., Dispositivos Intrauterinos, Planificación Familiar, Anticoncepción, Educación Sexual, Periodo Posparto.

## Abstract

**Objectives:**

to evaluate the insertion of the copper Intrauterine Device in the immediate postpartum period, considering side effects, pain intensity, information received, partner involvement, satisfaction, expulsion rate, and continued use.

**Methods:**

a prospective longitudinal study involving 117 women who had a copper Intrauterine Device inserted in the immediate postpartum period at a public maternity hospital in São Paulo, Brazil. Data were collected in the rooming-in unit through an online questionnaire.

**Results:**

a significant portion of the women did not receive information about advantages (31.8%) or side effects (42.4%). After six months, 37.5% had discontinued use due to expulsion (9.4%) or removal (28.1%). Side effects were reported by 60% of the women. Despite high satisfaction rates (85.3% at insertion and 75.0% at six months), the majority (60%) expressed a desire for a different contraceptive method.

**Conclusions:**

inadequate reproductive counseling may affect the continued use of intrauterine devices and influence contraceptive choices in the postpartum period.

## RESUMEN

**Objetivos:**

evaluar la inserción del Dispositivo Intrauterino de cobre en el posparto inmediato, considerando los efectos secundarios, la intensidad del dolor, la información recibida, la participación del acompañante, la satisfacción, la tasa de expulsión y la continuidad del uso.

**Métodos:**

Estudio longitudinal prospectivo con 117 mujeres que se colocaron el Dispositivo Intrauterino de cobre en el posparto inmediato en una maternidad pública de São Paulo, Brasil. Los datos se recopilaron en el alojamiento conjunto mediante un cuestionario en línea.

**Resultados:**

Una proporción significativa de mujeres no recibió información sobre las ventajas (31,8%) ni sobre los efectos secundarios (42,4%). A los seis meses, el 37,5% había interrumpido el uso, ya sea por expulsión (9,4%) o por retiro voluntario (28,1%). El 60% de las mujeres reportó efectos secundarios. A pesar de la alta satisfacción (85,3% en el momento de la inserción y 75,0% a los seis meses), la mayoría (60%) deseaba otro método anticonceptivo.

**Conclusión:**

La falta de asesoramiento reproductivo adecuado puede afectar la continuidad del uso del DIU e influir en la elección del

método anticonceptivo en el posparto.

## INTRODUCTION

In 2012, the Ministry of Health (MS) recognized the importance of initiatives aimed at women’s health during the postpartum period, highlighting the need for guidance on contraception during this time and unrestricted access to contraceptive methods as fundamental elements for the realization of sexual and reproductive rights^(1)^. In response to this need, the MS launched the Apice On Project in 2017, which included among its pillars the provision of the copper Intrauterine Device (IUD) during the immediate postpartum period and post-abortion care in public maternity hospitals across Brazil^(2)^.

Despite government incentives, high efficacy, and good acceptance - confirmed by user satisfaction and low discontinuation rates^(3-5)^- the IUD remains underutilized in some regions, especially in Latin America^(6)^. In Brazil, only 4.4% of women use the IUD^(7)^, a figure significantly lower than the global average of 16.8%^(8)^.

Regarding postpartum insertion of the method, it deserves special attention, particularly in areas where women face social, cultural, and/or geographic barriers to accessing services that offer contraceptive methods. According to Whitaker and Chen^(9)^, inserting the IUD immediately postpartum ensures contraceptive protection before fertility resumes and overcomes access barriers by eliminating the need to return to a healthcare facility. Moreover, the immediate postpartum period represents a window of opportunity for contraception, as many women show greater motivation to start a method, insertion is logistically more convenient, and there is certainty that they are not pregnant^(10)^. For these reasons, this period is ideal for IUD insertion and is an excellent strategy to reduce unintended pregnancies, increase interpregnancy intervals, and improve maternal and infant mortality indicators^(11)^.

Despite the numerous benefits of the IUD and the fact that it does not increase the risk of postpartum complications, one factor that may influence the decision-making process still persists: expulsion. It is known that when inserted immediately postpartum, the IUD has a higher expulsion rate (10-40%) compared to insertion six weeks after delivery (6%)^(12-14)^. However, studies have shown that if the expelled IUD is immediately replaced, the continuation rate and satisfaction with the method remain high, both six months and one year after delivery^(15,16)^. Therefore, the benefits of effective immediate postpartum contraception may outweigh the disadvantage of the increased risk of expulsion.

However, in Brazil, the insertion of the IUD during the immediate postpartum period remains underexplored, and most studies focus exclusively on expulsion rates, without addressing other relevant aspects of its use.

Given this gap, this study aims to expand knowledge about women’s experiences with IUD insertion in the immediate postpartum period, addressing not only clinical aspects but also factors related to the information received, the support provided, and the continuity of method use.

## OBJECTIVES

To evaluate the insertion of the copper intrauterine device (IUD) in the immediate postpartum period, considering side effects, pain intensity, the information provided by healthcare professionals, and the involvement of the companion in the process, as well as to assess user satisfaction, expulsion rate, and continuity of method use six months after insertion.

## METHODS

### Ethical aspects

The study was approved by Research Ethics Committees of the School of Arts, Sciences, and Humanities of the University of São Paulo (CAAE 25484619.7.0000.5390) and the Leonor Mendes de Barros Maternity Hospital (CAAE 25484619.7.3001.0063), and followed all ethical principles established in Resolution No. 466/2012 of the *Conselho Nacional de Saúde* (Brazilian National Health Council). Informed consent was obtained from all individuals involved in the study through an online process.

### Design, period and study location

This is a longitudinal prospective study conducted between June and November 2021 at a public maternity hospital in the municipality of São Paulo, Brazil. The study follows the guidelines of the Strengthening the Reporting of Observational Studies in Epidemiology (STROBE), recommended for observational studies in epidemiology by the Enhancing the QUAlity and Transparency Of health Research (EQUATOR) network.

### Population or sample, inclusion and exclusion criteria

The sample was non-probabilistic and consisted of women aged 18 years or older who had vaginal, cesarean, or instrumental deliveries (forceps or vacuum extractor) and underwent copper intrauterine device (IUD) insertion during the immediate postpartum period. Inclusion criteria were age 18 or older, copper IUD insertion in the immediate postpartum period, internet access, availability of a phone number or email address, and spoken comprehension of the Portuguese language.

Considering that the healthcare facility where the study was conducted performs an average of 350 deliveries and 32 postpartum IUD insertions per month, it was estimated that approximately 130 insertions could be carried out over a four-month period, resulting in an expected sample of 130 interviews. However, during the data collection period, the COVID-19 pandemic significantly affected the facility’s operations, reducing the availability of supplies and the number of trained professionals for IUD insertion. These limitations led to a lower number of insertions and, consequently, to the inclusion of 117 women invited to participate in the study, of whom 116 agreed, forming the final sample.

### Study protocol

The study was conducted in two phases:

1. Initial Data Collection

During the postpartum hospitalization period, in the rooming-in unit, eligible women who had undergone IUD insertion were individually approached by researchers with degrees in health sciences. The researchers explained the study objectives and invited the patients to participate. Those who agreed completed an online questionnaire via the Google Forms platform, accessed through a tablet provided by the researchers.

2. Six-Month Follow-Up (via WhatsApp)

Six months after IUD insertion, participants were invited via the WhatsApp application to complete a second questionnaire through Google Forms. In cases of non-response, three follow-up attempts were made via WhatsApp, followed by contact through email. In total, 32 women completed the second phase of the study.

### Analysis of the results and statistics

Data analysis was performed using Stata 14.2 software (https://www.stata.com) in two stages:

1. Descriptive analysis, presenting variables in absolute numbers, proportions, means, and standard deviations, covering sociodemographic profile, sexual, reproductive, and contraceptive behavior, obstetric history, information received about the IUD during hospitalization, and specific characteristics of IUD insertion.
2. Outcome analysis after six months, also expressed in absolute numbers and proportions, including: sexual and contraceptive behavior after hospital discharge, and attitudes and practices related to the IUD six months after insertion.

Missing data were handled through complete case analysis, with no value imputation.

## RESULTS

The sociodemographic characteristics of the women are presented in Table 1. The participants had a mean age of 28 years (SD = 6.0), ranging from 15 to 43 years. Regarding socioeconomic status, most belonged to classes C-D/E, according to the Brazil Economic Classification Criteria^1 7^. In terms of sexual behavior, the average age at first sexual intercourse was 16 years (SD = 1.66), and the majority reported having had between three and four lifetime sexual partners (35.3%) (data not shown in table). As for reproductive behavior, the first pregnancy occurred at an average age of 21 years (SD = 4.9). Most women were multigravida (three or more pregnancies) (47.4%) and multiparous (three or more births) (39.7%) (Table 1). Of the 116 participants, the majority received prenatal care through the public health system (95.7%), and 68.1% had not planned their pregnancy. Regarding the most recent delivery, most women had a vaginal birth (62.0%) between 37 and 39 weeks of gestation (56.9%) (data not shown in table).

**Table 1:** Sociodemographic and Reproductive Characteristics of Women Who Underwent Immediate Postpartum Intrauterine Device Insertion at a Public Maternity Hospital, São Paulo, Brazil, 2021 (continues)

Variables	n	%
**Age**		
≤ 24 years	34	29.3
25-34 years	61	52.6
≥ 35 years	21	18.1
**Race/Ethnicity**		
White	43	37.1
Brown (Mixed-race)	50	43.1
Black	19	16.4
Other (Asian)	04	3.4
**Educational Level**		
Illiterate - Incomplete Primary Education	14	12.0
Completed Primary Education - Incomplete Secondary Education	19	16.4
Completed Secondary Education - Incomplete Higher Education	62	53.5
Completed Higher Education	21	18.1
**Religion**		
None	18	15.5
Roman Catholic	36	31.1
Evangelical	52	44.8
Other (including Spiritism, Judaism, Jehovah’s Witnesses, Buddhism, and Islam)	10	8.6
**Socioeconomic level**		
A/B	14	12.1
C-D/E	102	87.9
**Relationship (married or in a stable union)**		
No	15	12.9
Yes	101	87.1
**Lives with the partner**		
No	16	13.8
Yes	100	86.2
**Number of pregnancies (including current)**		
One	27	23.3
Two	34	29.3
Three or more	55	47.4
**Number of deliveries (including current)**		
One	28	24.1
Two	42	36.2
Three or more	46	39.7
**Total**	**116**	**100.0**

Regarding the information provided by healthcare professionals about the IUD (Table 2), most women had already heard about postpartum IUD use (71.5%). In the hospital, the majority received information about the possibility of IUD insertion during labor (56.0%), primarily from physicians (50.0%). However, notably, 42.4% of the women were not informed about potential side effects of the method, and more than half (52.9%) did not receive guidance on the possibility of using other contraceptive methods besides the IUD during the postpartum period.

**Table 2:** Provision of Information About the Intrauterine Device to Women Who Underwent Postpartum Insertion at a Public Maternity Hospital, São Paulo, Brazil, 2021

Variables	n	%
**Had heard about the IUD in the postpartum period**		
No	33	28.5
Yes	83	71.5
**At the hospital, moment when the participant first heard about the postpartum IUD (n = 263)**		
During hospitalization	74	28.1
During labor	65	56.0
During delivery	62	17.2
After delivery	62	14.7
**At the hospital, type of healthcare professional who provided information about the postpartum IUD**		
Physician	58	50.0
Nurse	25	21.6
Midwife	21	18.1
Other professional	12	10.3
**At the hospital, received specific information about the IUD from a healthcare professional**		
No	31	26.7
Yes	85	73.3
**Received information on how the IUD prevents pregnancy (n = 85)**		
No	05	5.9
Yes	80	94.1
**Received information on advantages and disadvantages of the postpartum IUD (n = 85)**		
No	27	31.8
Yes	58	68.2
**Received information on the possibility of using other contraceptive methods (n = 85)**		
No	45	52.9
Yes	40	47.1
**Received information on possible side effects of the postpartum IUD (n = 85)**		
No	36	42.4
Yes	49	57.6
**Total**	**116**	**100**

*IUD - Intrauterine Device.*

Concerning the IUD insertion itself, only a small proportion of women (34.5%) had the procedure performed by nurses or midwives. Overall, slightly more than one-quarter of the participants reported experiencing pain after insertion. Among these, the pain was most commonly described as moderate (71.0%). Although the majority of women reported being satisfied with postpartum IUD insertion (85.3%), 61.2% stated they would have preferred a different contraceptive method, with tubal ligation being the most cited alternative (59.2%) (Table 3).

**Table 3:** Immediate Postpartum Intrauterine Device Insertion in Women Assisted at a Public Maternity Hospital, São Paulo, Brazil, 2021

Variables	n	%
**Type of healthcare professional who performed postpartum IUD^1^ insertion**		
Physician	76	65.5
Nurse	07	6.0
Midwife	33	28.5
**Pain after postpartum IUD insertion**		
No	85	73.3
Yes	31	26.7
**Intensity of pain during postpartum IUD insertion (n=31)**		
Mild	06	19.3
Moderate	22	71.0
Severe	02	6.5
Very severe	01	3.2
**Satisfaction with postpartum IUD insertion**		
No	0	0.0
Yes	99	85.3
Somewhat	17	14.7
**Would choose another method instead of the IUD**		
No	45	38.8
Yes	71	61.2
**Type of method preferred instead of the IUD (n=71)**		
Tubal ligation	42	59.2
Oral contraceptive pill	08	11.3
Implant	06	8.5
Injectable contraceptive	05	7.0
Other method2	10	14.0
**Presence of companion during IUD counseling at the hospital**		
No	20	17.2
Yes	96	82.8
**Type of companion present during IUD counseling (n=96)**		
Partner	73	76.0
Mother	14	14.6
Other person (sister, cousin, neighbor)	09	9.4
**Discussed the IUD with companion and whether they assisted in the decision-making process (n=96)**		
Did not discuss it; therefore, the companion did not assist in choosing the method	17	17.7
Yes, discussed it, but I decided alone; the companion did not assist in choosing the method	30	31.2
Yes, discussed it and the companion assisted in choosing the method	49	51.0
**Total**	**116**	**100.0**

IUD - Intrauterine Device;

* Other method: contraceptive patch, male condom, vasectomy, fertility awareness-based methods.

Among the women who were accompanied during the IUD counseling at the hospital (n = 96), the majority were accompanied by their partner (76.0%). However, in 49% of cases, the companion did not participate in the decision-making process regarding IUD insertion (Table 3).

At the six-month follow-up, only 32 women responded to the questionnaire. Although the non-response rate was higher than expected (27.6%), the distribution of losses was uniform, suggesting that the attrition was random (p = 0.345). Among the 32 women who participated in the second phase of the study, the majority (62.5%) were still using the IUD. Of those who attended a follow-up consultation (n = 29), 89.7% underwent ultrasound to verify the device’s positioning (Table 4). Among these, 80.8% had an ultrasound within the first 30 days after insertion, and 19.2% between the second and sixth month. In 34.6% of cases, the IUD was found to be improperly positioned (data not shown in table).

**Table 4:** Follow-Up of Intrauterine Device Insertion After Six Months of Use Among Women Assisted at a Public Maternity Hospital, São Paulo, Brazil, 2021

Variables	n	%
**IUD use at six months**		
No	12	37.5
Yes	20	62.5
**Ultrasound examination to verify IUD positioning (n=29)**		
No	03	10.3
Yes	26	89.7
**IUD intentionally removed**		
No	23	71.9
Yes	09	28.1
**IUD expulsion**		
No	29	90.6
Yes	03	9.4
**Side effects during the first six months**		
No	13	40.6
Yes	19	59.4
**Satisfaction with IUD use during the first six months**		
Dissatisfied	04	12.5
Slightly satisfied	04	12.5
Moderately satisfied	10	31.2
Very satisfied	14	43.8
**Would choose another method instead of the IUD**		
No	12	37.5
Yes	20	62.5
**Type of method preferred instead of the IUD (n=20)**		
Injectable	14	70.0
Implant	04	20.0
Oral contraceptive pill	02	10.0
**Deprivation of companion’s presence during the postpartum period**		
No, I was deprived of my companion’s presence during the postpartum period	17	53.1
Yes, but not during the decision-making and/or IUD insertion	11	34.4
Yes, including during the decision-making and/or IUD insertion	04	12.5
**Total**	**32**	**100.0**

*IUD - Intrauterine Device.*

Among the women who discontinued use of the method after six months, three experienced IUD expulsion. Additionally, nine women chose or were advised to remove the IUD due to medical recommendation, poor adaptation to the method, or improper positioning (data not shown in table).

Most women reported side effects during the first six months of IUD use (60%) (Table 4). The most frequently reported effects (multiple responses allowed) were increased menstrual flow (56.3%), intensified menstrual cramps (50.0%), irregular bleeding (37.5%), and weight gain (12.5%) (data not shown in table). Despite 75.0% of women reporting moderate to high satisfaction with the IUD after six months of use, the majority (62.5%) stated they would have preferred a different method, with injectable contraception being the preferred alternative for 70.0% of them.

## DISCUSSION

The study revealed important findings regarding immediate postpartum copper IUD insertion. Most insertions occurred after vaginal delivery and were performed by physicians, which may be related to the higher proportion of vaginal births during the data collection period, the professionals’ preference and greater confidence in performing the procedure in this context, and possible post-cesarean complications that prevent insertion. The limited participation of nurses and midwives in the process may reflect organizational barriers, such as the medical exclusivity in performing IUD insertions and the lack of training for other professionals, thereby restricting the potential of the multidisciplinary team in reproductive planning^(18,19)^.

Another critical finding was the deficiency in information delivery about the IUD to women. Approximately one-third of participants did not receive adequate guidance on the advantages, disadvantages, and side effects of the method, which may have directly impacted its continued use and influenced the preference for other contraceptive options. It was observed that, for many women, the first contact with information about the IUD occurred only during labor or delivery - moments that are potentially inappropriate for informed reproductive decision-making. In this regard, it is essential to emphasize that labor and delivery are not considered ideal times for offering the IUD, as these are potentially disorganizing events for women, who are in a state of emotional and physical vulnerability. Consequently, reproductive autonomy may be subject to various mechanisms of contraceptive coercion^(20)^.

Regarding users’ perceptions of the device insertion, just over one-quarter of women reported moderate pain, a finding similar to that observed in another study conducted with postpartum Brazilian adolescents^(21)^. However, it is not possible to determine whether the reported pain was due to the insertion itself or to uterine involution.

An important aspect that deserves attention is user satisfaction with the IUD: 31.2% of women reported being satisfied and 43.8% very satisfied, which contrasts with the limitations in counseling and the lack of opportunity to choose other contraceptive methods. Many participants expressed a preference for alternatives, such as tubal ligation or injectable contraceptives, at different points in time. The initial preference for tubal ligation may be associated with the recent childbirth experience and the desire to end the reproductive phase, while the later preference for short-term methods, such as injectables, suggests a search for greater control and flexibility. These findings, combined with the fact that a significant proportion of women did not receive adequate counseling about the IUD and that more than half experienced side effects, underscore the need to strengthen access to information and postpartum contraceptive counseling. More qualified strategies may foster user trust, motivation, and satisfaction, thereby promoting continued use of the method^(22,23)^. From this perspective, although government initiatives promote the copper IUD as a highly effective method for postpartum and post-abortion contraception, the restricted offer of a single method and the persuasive approach to its acceptance-often linked to the achievement of targets by healthcare professionals-may result in a disproportionate emphasis on one method (in this case, the IUD). This restricts women’s ability to choose and limits their autonomy, potentially constituting practices of contraceptive coercion^(24,25)^.

Furthermore, it is important to critically examine the lack of desire for the IUD, as the preference for tubal ligation in the Brazilian context reflects not only social constructs surrounding motherhood but also negative experiences with reversible methods, misinformation, concerns about side effects, and the absence of qualified dialogue with healthcare professionals. Qualitative studies could provide deeper insight into the subjective and contextual factors that influence these choices.

In the context of contraceptive care, partner involvement in the decision-making process remains a challenge, as evidenced in this study. For many women, contraceptive practice is perceived as an individual responsibility, without shared participation from their partner, reflecting historically constructed gender inequalities in the field of reproduction^(26-28)^. In-depth qualitative studies could contribute to a better understanding of this decision-making process, exploring not only the reasons behind the absence of partner involvement but also the factors that lead some women to prefer making contraceptive choices independently.

Regarding the six-month follow-up, the IUD expulsion rate was 9.4%, lower than reported in other national studies^(21,29)^, while the removal rate was higher than in previous research^(18,30)^. These variations may be attributed to several factors, including differences in population profiles, sample sizes, insertion techniques, and follow-up strategies. Continuation of IUD use at the end of the study was also lower than in other investigations^(18,29)^, suggesting that some women may have been coerced into using the method, as previously discussed.

A particularly noteworthy finding was the excessive use of transvaginal ultrasounds to verify IUD positioning, a practice not aligned with the guidelines of the Brazilian Ministry of Health^(31)^. Finally, the restriction of companion presence during the postpartum period amid the pandemic highlighted additional vulnerabilities related to sexual and reproductive rights and access to family planning services.

These findings should be interpreted in light of the care model in place at the investigated health service, which continues to prioritize childbirth-centered interventions over ongoing reproductive planning efforts. Weak counseling strategies, the absence of protocols integrating immediate postpartum care, and limited multidisciplinary engagement may have influenced both initial IUD uptake and its discontinuation. Strengthening the educational and health-promoting role of nursing and other professionals is essential to ensure woman-centered care, with comprehensive and unbiased information.

### Study limitations

This study presents several limitations, including a sample restricted to a single research site and a reduced number of participants, reflecting the challenges of data collection during the COVID-19 pandemic. The shortage of supplies and the reduced availability of qualified professionals for IUD insertion during this period may have limited access to the method to a specific subgroup of women, potentially introducing selection bias and affecting the generalizability of the findings. Nevertheless, the analyzed population included all postpartum women who received an IUD at the hospital, which lends relevance to the results.

Another significant limitation concerns the high loss to follow-up at six months (72%), with only 32 women completing the second phase of the study. Although strategies were employed to minimize these losses, such as multiple contact channels and periodic reminders, they may have influenced the outcome analysis. It is possible that some of the losses were related to discontinuation of use or dissatisfaction with the method, which could introduce bias and overestimate the continuation rate. While the data suggest that losses were evenly distributed across the sociodemographic characteristics assessed, the influence of unmeasured factors cannot be ruled out. Additionally, the use of a non-probabilistic sample limits the generalizability of the findings. Finally, the descriptive nature of the analysis does not allow for statistically significant associations between the variables studied. Despite these limitations, the study is innovative and highlights important gaps, such as the lack of adequate information and biased counseling regarding immediate postpartum IUD use by healthcare professionals.

### Contributions to Nursing, Health, and Public Policy

The findings of this study may support the development and implementation of public policies focused on postpartum sexual and reproductive health, with an emphasis on the provision of adequate information. The free availability of contraceptive methods through the *“SUS”*, without proper counseling, limits full access to reproductive rights. Furthermore, the minimal participation of nursing professionals in the studied setting highlights the need to revisit their role in reproductive planning. It is essential to strengthen the role of nursing as a health educator and promoter, adopting a womanand couple-centered approach that ensures qualified guidance and supports informed decision-making.

## CONCLUSIONS

The study revealed weaknesses in institutional strategies for postpartum reproductive planning and counseling, with approximately half of the women reporting that they would have preferred a method other than the IUD. Many did not receive sufficient information about side effects, benefits, disadvantages, or alternative contraceptive options. Notably, for more than half of the participants, the first contact with information about the IUD occurred only during labor or delivery-moments when reproductive autonomy may be compromised. These findings underscore the urgent need for policies and practices that expand and improve contraceptive counseling during the postpartum period, promoting informed choices and respecting each woman’s individual circumstances. Such efforts are essential to reducing health inequities and social disparities.

## Data Availability

The research data are available only upon request.
