# Supplementary material for: Use of copper intrauterine devices in the immediate postpartum period: a prospective longitudinal study
Source: Rev Bras Enferm. 2026 Jul 6;79:e20250153. doi: 10.1590/0034-7167-2025-0153 (PMC13336227; doi:10.1590/0034-7167-2025-0153)
Supplement: Translation

# Translation: Uso de dispositivos intrauterinos de cobre no pós-parto imediato: estudo longitudinal prospectivo

**DOI:** 10.1590/0034-7167-2025-0153pt

**Authors:** Christiane Borges do Nascimento Chofakian, Giovana Nonato, Raphaela Romão Teixeira de Souza, Rosemeire Sartori de Albuquerque, Alícia Rafaelly Vilefort Sales, Ana Luiza Vilela Borges

**Affiliations:** I Universidade de São Paulo. São Paulo, São Paulo, Brasil

**Keywords:** Dispositivos Intrauterinos, Planejamento Familiar, Anticoncepção, Educação Sexual, Período Pós-Parto.

## Abstract

**Objetivos:**

avaliar a inserção do dispositivo intrauterino de cobre no pós-parto imediato, considerando efeitos colaterais, intensidade da dor, informações recebidas, participação do acompanhante, satisfação, taxa de expulsão e continuidade do uso.

**Métodos:**

estudo longitudinal-prospectivo com 117 mulheres que inseriram o dispositivo intrauterino de cobre no pós-parto imediato em uma maternidade pública de São Paulo, Brasil. Os dados foram coletados no alojamento conjunto por questionário online.

**Resultados:**

uma parcela significativa das mulheres não recebeu informações sobre vantagens (31,8%) e efeitos colaterais (42,4%). Após seis meses, 37,5% haviam interrompido o uso, por expulsão (9,4%) ou remoção (28,1%). Efeitos colaterais foram relatados por 60% das mulheres. Apesar da satisfação elevada (85,3% na inserção e 75,0% aos seis meses), a maioria das mulheres (60%) desejava outro método contraceptivo.

**Conclusões:**

a falta de aconselhamento reprodutivo adequado pode impactar a continuidade do uso do dispositivo intrauterino e influenciar a escolha contraceptiva no pós-parto.

## INTRODUÇÃO

O Ministério da Saúde (MS), em 2012, reconheceu a importância das ações voltadas para a saúde da mulher durante o pós-parto, destacando a necessidade de orientações sobre contracepção neste período e o acesso irrestrito aos métodos como elementos fundamentais para a efetivação dos direitos sexuais e reprodutivos^(1)^. Diante dessa necessidade, destaca-se a iniciativa do MS em 2017, por meio do Projeto Apice On, que teve como um dos pilares a oferta do Dispositivo Intrauterino (DIU) de cobre no puerpério imediato e no pós-abortamento em maternidades públicas do Brasil^(2)^.

Apesar dos incentivos governamentais, da alta eficácia e da boa aceitação - ratificado pela satisfação e baixa descontinuidade do método^(3-5)^ - o DIU permanece subutilizado em algumas regiões, sobretudo na América Latina^(6)^. No Brasil apenas 4,4% das mulheres utilizam o DIU^(7)^, dado muito inferior ao global (16,8%)^(8)^.

No que concerne à inserção do método no pós-parto, esta merece especial destaque, sobretudo em locais onde as mulheres enfrentam limitações sociais, culturais e/ou geográficas para acessar serviços que disponibilizam métodos contraceptivos. Conforme Whitaker e Chen^(9)^, a inserção do DIU no pós-parto imediato garante proteção contraceptiva antes da retomada da fertilidade e supera barreiras de acesso ao evitar a necessidade de retorno ao serviço de saúde. Além disso, o período pós-parto imediato representa uma janela de oportunidade para a anticoncepção, uma vez que muitas mulheres demonstram maior motivação para iniciar um método, a inserção ser logisticamente mais conveniente e haver a garantia de que não estão grávidas^(10)^. Por estes motivos, este período é oportuno para inserção do DIU, sendo uma excelente estratégia para reduzir gravidezes não intencionais, aumentar os intervalos intergestacionais e melhorar os indicadores de mortalidade materna e infantil^(11)^.

Apesar dos inúmeros benefícios do DIU e do fato de não aumentar o risco de complicações no pós-parto, um fator que pode interferir no processo decisório pelo método ainda persiste: a expulsão. Sabe-se que, quando inserido no pós-parto imediato, o DIU possui um índice de expulsão maior (10-40%) em relação à inserção seis semanas após o parto (6%)^(12-14)^. Porém, estudos observaram que, se houver substituição imediata do DIU expelido, a taxa de continuidade e a satisfação com o método são elevadas, tanto seis meses quanto um ano após o parto^(15,16)^. Portanto, os benefícios da efetividade da contracepção imediata após o parto podem compensar a desvantagem do risco de expulsão aumentado.

No entanto, no Brasil, a inserção do DIU no pós-parto imediato ainda é pouco explorada, e a maioria dos estudos concentra-se exclusivamente nas taxas de expulsão, sem abordar outros aspectos relevantes sobre seu uso.

Considerando essa lacuna, este estudo busca ampliar o conhecimento sobre a experiência das mulheres com a inserção do DIU no pós-parto imediato, abordando não apenas aspectos clínicos, mas também fatores relacionados à informação recebida, ao suporte oferecido e à continuidade do uso do método.

## OBJETIVOS

Avaliar a inserção do dispositivo intrauterino de cobre no pós-parto imediato, considerando os efeitos colaterais, a intensidade da dor, as informações fornecidas pelos profissionais de saúde e a participação do acompanhante no processo, bem como verificar a satisfação das usuárias, a taxa de expulsão e a continuidade do uso do método após seis meses da inserção.

## MÉTODOS

### Aspectos éticos

O estudo foi aprovado pelo Comitê de Ética em Pesquisa da Escola de Artes, Ciências e Humanidades da Universidade de São Paulo (CAAE 25484619.7.0000.5390) e do Hospital Maternidade Leonor Mendes de Barros (CAAE 25484619.7.3001.0063), e seguiu todos os preceitos éticos estabelecidos na Resolução nº 466/2012 do Conselho Nacional de Saúde. O Consentimento Livre e Esclarecido foi obtido de todos os indivíduos envolvidos no estudo por meio online.

### Desenho, período e local do estudo

Trata-se de um estudo longitudinal-prospectivo, conduzido entre junho e novembro de 2021 em uma maternidade pública do município de São Paulo, Brasil. O estudo segue as diretrizes da *Strengthening the Reporting of Observational Studies in Epidemiology* (STROBE), recomendada para estudos observacionais em epidemiologia pela rede *Enhancing the QUAlity and Transparency Of health Research* (EQUATOR).

### População ou amostra, critérios de inclusão e exclusão

A amostra foi não probabilística e composta por mulheres com 18 anos ou mais, que tiveram parto vaginal, cesárea ou instrumental (fórceps ou vácuo-extrator) e que realizaram a inserção do dispositivo intrauterino (DIU) de cobre no pós-parto imediato. Foram considerados critérios de inclusão: idade igual ou superior a 18 anos, inserção do DIU de cobre no pós-parto imediato, acesso à internet e disponibilidade de contato telefônico ou endereço eletrônico (e-mail), e compreensão falada da língua portuguesa.

Considerando que, no serviço de saúde onde o estudo foi desenvolvido, ocorrem, em média, 350 nascimentos/mês e 32 inserções de DIU/mês no pós-parto, estimou-se que, em um período de quatro meses, poderiam ser realizadas aproximadamente 130 inserções, resultando em uma amostra esperada de 130 entrevistas. No entanto, durante o período de coleta, a pandemia de COVID-19 impactou significativamente o funcionamento do serviço, com redução na disponibilidade de insumos e no número de profissionais capacitados para a inserção do DIU. Essas limitações resultaram em um número menor de inserções e, consequentemente, na inclusão de 117 mulheres convidadas a participar do estudo, das quais 116 aceitaram, compondo a amostra final.

### Protocolo do estudo

O estudo foi conduzido em dois momentos:

1. Coleta inicial

Durante o período de internação pós-parto, no alojamento conjunto, as mulheres elegíveis que realizaram a inserção do DIU foram abordadas individualmente por pesquisadoras graduadas na área da saúde, que explicaram os objetivos do estudo e convidaram as pacientes a participar. As que aceitaram responderam um questionário *online*, via plataforma *Google Forms*, acessado por meio de um tablet disponibilizado pelas pesquisadoras.

2. Acompanhamento após seis meses (seguimento via *WhatsApp*)

Seis meses após a inserção do DIU, as participantes foram convidadas, por meio do aplicativo *WhatsApp*, a responder um segundo questionário via *Google Forms*. Na ausência de respostas, foram realizadas três tentativas de contato pelo *WhatsApp* e, posteriormente, foi realizado contato via e-mail. No total, 32 mulheres completaram a segunda etapa da pesquisa.

### Análise dos resultados e estatística

A análise dos dados foi realizada no software Stata 14.2 (https://www.stata.com), em duas etapas:

1. Análise descritiva, com apresentação das variáveis em números absolutos, proporções, médias e desvio-padrão, abordando o perfil sociodemográfico, comportamento sexual, reprodutivo e contraceptivo, histórico obstétrico, informações recebidas sobre o DIU durante a internação e características específicas da inserção do DIU.
2. Análise dos desfechos após seis meses, também expressa em números absolutos e proporções, incluindo: comportamento sexual e contraceptivo após a alta hospitalar e atitudes e práticas relacionadas ao DIU seis meses após a inserção.

Os dados ausentes foram tratados por meio da análise de casos completos, sem imputação de valores.

## RESULTADOS

A caracterização sociodemográfica das mulheres encontra-se na Tabela 1. As mulheres do estudo tinham, em média, 28 anos de idade (DP = 6,0), variando entre 15 e 43 anos. Em relação à condição socioeconômica, grande parte pertencia às classes C-D/E, segundo o Critério de Classificação Econômica Brasil^(17)^. No que se refere ao comportamento sexual, a idade média de início da relação sexual foi de 16 anos (DP = 1,66), e a maioria das mulheres relatou ter tido entre três e quatro parceiros ao longo da vida (35,3%) (dados não apresentados em tabela). Quanto ao comportamento reprodutivo, a primeira gravidez ocorreu, em média, aos 21 anos (DP = 4,9). A maioria das mulheres era multigesta (três ou mais gestações) (47,4%) e multípara (três ou mais partos) (39,7%) (Tabela 1). Das 116 participantes, a maioria realizou o pré-natal no serviço público (95,7%), e 68,1% não planejaram a gravidez. Em relação ao último parto, a maioria das mulheres teve parto vaginal (62,0%) entre 37 e 39 semanas de gestação (56,9%) (dados não apresentados em tabela).

**Tabela 1:** Características sociodemográficas e reprodutivas de mulheres que inseriram dispositivo intrauterino no pós-parto imediato em uma maternidade pública, São Paulo, Brasil, 2021

Variáveis	n	%
**Idade**		
≤ 24 anos	34	29,3
25-34 anos	61	52,6
≥ 35 anos	21	18,1
**Raça/Etnia**		
Branca	43	37,1
Parda	50	43,1
Preta	19	16,4
Outra (amarela)	04	3,4
**Escolaridade**		
Analfabeto - Fundamental Incompleto	14	12,0
Fundamental Completo - Ensino Médio Incompleto	19	16,4
Ensino Médio Completo - Superior Incompleto	62	53,5
Superior Completo	21	18,1
**Religião**		
Nenhuma	18	15,5
Católica Romana	36	31,1
Evangélica	52	44,8
Outras (Espiritismo, Judaísmo, Testemunha de Jeová, Budismo e Islamismo)	10	8,6
**Nível Socioeconômico**		
A/B	14	12,1
C-D/E	102	87,9
**Relacionamento (casada ou união estável)**		
Não	15	12,9
Sim	101	87,1
**Mora com o parceiro(a)**		
Não	16	13,8
Sim	100	86,2
**Número de gestações (contando a atual)**		
Uma	27	23,3
Duas	34	29,3
Três ou mais	55	47,4
**Número de partos (contando o atual)**		
Um	28	24,1
Dois	42	36,2
Três ou mais	46	39,7
**Total**	**116**	**100,0**

Em relação às informações sobre o DIU fornecidas pelos profissionais de saúde (Tabela 2), a maioria das mulheres já havia ouvido falar sobre o DIU no pós-parto (71,5%). No hospital, a maioria delas obteve informações sobre a possibilidade de inserção do DIU durante o trabalho de parto (56,0%) por meio do médico(a) (50,0%). No entanto, surpreendentemente, 42,4% das mulheres não foram informadas sobre os possíveis efeitos colaterais do método, e mais da metade (52,9%) não recebeu orientações sobre a possibilidade de utilizar outros métodos além do DIU no pós-parto.

**Tabela 2:** Oferta de informações sobre o dispositivo intrauterino às mulheres que inseriram este método em uma maternidade pública, São Paulo, Brasil, 2021

Variáveis	n	%
**Havia ouvido falar sobre o DIU no pós-parto**		
Não	33	28,5
Sim	83	71,5
**No hospital, momento em que ouviu falar sobre o DIU no pós-parto (n=263)**		
Na internação	74	28,1
Durante o trabalho de parto	65	56,0
Durante o parto	62	17,2
Após o parto	62	14,7
**No hospital, tipo de profissional de saúde que falou sobre o DIU no pós-parto**		
Médico(a)	58	50,0
Enfermeiro(a)	25	21,6
Obstetriz	21	18,1
Outro profissional	12	10,3
**No hospital, recebeu informações específicas sobre o DIU pelo profissional de saúde**		
Não	31	26,7
Sim	85	73,3
**Recebeu informações sobre como o DIU previne uma gravidez (n = 85)**		
Não	05	5,9
Sim	80	94,1
**Recebeu informações sobre vantagens e desvantagens do DIU no pós-parto (n=85)**		
Não	27	31,8
Sim	58	68,2
**Recebeu informações sobre a possibilidade de usar outros métodos (n = 85)**		
Não	45	52,9
Sim	40	47,1
**Recebeu informações sobre os efeitos colaterais do DIU no pós-parto (n = 85)**		
Não	36	42,4
Sim	49	57,6
**Total**	**116**	**100**

*DIU - Dispositivo Intrauterino.*

Com relação à inserção do DIU, apenas uma pequena parte das mulheres (34,5%) teve o procedimento realizado por enfermeiras ou obstetrizes. No total, um pouco mais de um quarto das mulheres relataram ter sentido dor após a inserção do método. Dentre estas, a dor foi considerada como moderada pela maioria (71,0%). Embora a maioria das mulheres relatou ter ficado satisfeita quanto à inserção do DIU no pós-parto (85,3%), 61,2% afirmaram que escolheriam outro método ao invés do DIU, sendo a laqueadura a preferência de 59,2% dessas mulheres (Tabela 3).

**Tabela 3:** Inserção do dispositivo intrauterino no pós-parto imediato em mulheres assistidas em uma maternidade pública, São Paulo, Brasil, 2021

Variáveis	n	%
**Tipo de profissional que inseriu o DIU no pós-parto**		
Médico(a)	76	65,5
Enfermeiro(a)	07	6,0
Obstetriz	33	28,5
**Dor após inserção do DIU no pós-parto**		
Não	85	73,3
Sim	31	26,7
**Intensidade da dor ao inserir o DIU no pós-parto (n=31)**		
Fraca	06	19,3
Moderada	22	71,0
Forte	02	6,5
Muito Forte	01	3,2
**Satisfação quanto à inserção do DIU no pós-parto**		
Não	0	0,0
Sim	99	85,3
Mais ou menos	17	14,7
**Escolheria outro método ao invés do DIU**		
Não	45	38,8
Sim	71	61,2
**Tipo de método que escolheria ao invés do DIU (n=71)**		
Laqueadura	42	59,2
Pílula oral	08	11,3
Implante	06	8,5
Injetável	05	7,0
Outro método^*^	10	14,0
**Presença do acompanhante durante a oferta do DIU no hospital**		
Não	20	17,2
Sim	96	82,8
**Tipo de acompanhante na oferta do DIU (n=96)**		
Companheiro(a)	73	76,0
Mãe	14	14,6
Outra pessoa (irmã, prima, vizinha)	09	9,4
**Conversou com acompanhante sobre o DIU e este ajudou na decisão pela escolha do método (n=96)**		
Não conversei e, portanto, o acompanhante não ajudou a decidir sobre o método	17	17,7
Sim, conversei, mas eu decidi sozinha, o acompanhante não ajudou a decidir sobre o método	30	31,2
Sim, conversei e o acompanhante ajudou a decidir sobre o método	49	51,0
**Total**	**116**	**100,0**

DIU - Dispositivo Intrauterino;

* Outro método: adesivo, preservativo masculino, vasectomia, tabelinha.

Dentre as mulheres que estavam acompanhadas durante a oferta do DIU no hospital (n = 96), a maioria teve a presença do companheiro(a) (76,0%), mas em 49% dos casos, o acompanhante não participou da decisão sobre a inserção do DIU (Tabela 3)

No acompanhamento realizado aos seis meses, apenas 32 mulheres responderam ao questionário. Embora a taxa de não resposta tenha sido maior do que o esperado (27,6%), a distribuição das perdas foi uniforme, o que sugere que a perda foi aleatória (p = 0,345). Dentre as 32 mulheres que participaram da segunda etapa do estudo, a maioria (62,5%) continuava utilizando o DIU. Entre as mulheres que realizaram a consulta de retorno (n = 29), 89,7% fizeram ultrassonografia para verificar o posicionamento do dispositivo (Tabela 4). Destas, 80,8% realizaram a ultrassonografia nos primeiros 30 dias após a inserção, e 19,2% entre o segundo e o sexto mês após a inserção, sendo que em 34,6% dos casos, o DIU estava fora da posição adequada (dados não apresentados em tabela).

**Tabela 4:** Acompanhamento da inserção do dispositivo intrauterino aos seis meses de uso do método em mulheres assistidas em uma maternidade pública, São Paulo, Brasil, 2021

Variáveis	n	%
**Uso do DIU aos seis meses**		
Não	12	37,5
Sim	20	62,5
**Exame de ultrassonografia para verificar o posicionamento do DIU (n = 29)**		
Não	03	10,3
Sim	26	89,7
**DIU retirado intencionalmente**		
Não	23	71,9
Sim	09	28,1
**Expulsão do DIU**		
Não	29	90,6
Sim	03	9,4
**Efeitos colaterais nos primeiros seis meses**		
Não	13	40,6
Sim	19	59,4
**Satisfação com uso do DIU nos primeiros seis meses**		
Insatisfeita	04	12,5
Pouco satisfeita	04	12,5
Moderadamente satisfeita	10	31,2
Muito satisfeita	14	43,8
**Escolheria outro método ao invés do DIU**		
Não	12	37,5
Sim	20	62,5
**Tipo de método que escolheria ao invés do DIU (n=20)**		
Injetável	14	70,0
Implante	04	20,0
Pílula oral	02	10,0
**Privação da presença do acompanhante no pós-parto**		
Não fui privada da presença do meu acompanhante no pós-parto	17	53,1
Sim, mas não durante a decisão e/ou durante inserção do DIU	11	34,4
Sim, inclusive na decisão e/ou durante a inserção do DIU	04	12,5
**Total**	**32**	**100,0**

*DIU - Dispositivo Intrauterino.*

Entre as mulheres que não continuaram com o uso do método após seis meses, três tiveram expulsão do DIU. Além disso, nove mulheres optaram ou precisaram retirar o DIU (indicação do profissional de saúde, não adaptação ao método e posicionamento inadequado) (dados não apresentados em tabela).

A maioria das mulheres relatou efeitos colaterais nos primeiros seis meses de uso do DIU (60%) (Tabela 4). Os efeitos mais frequentes (questão que admitiu múltipla resposta) foram aumento do fluxo menstrual (56,3%), intensificação das cólicas menstruais (50,0%), sangramento irregular (37,5%) e aumento de peso (12,5%) (dados não apresentados em tabela). Apesar de 75,0% das mulheres relatarem satisfação moderada ou alta com o DIU, mesmo após seis meses de uso, a maioria (62,5%) preferiria ter escolhido outro método, sendo o método injetável o de preferência para 70,0% delas.

## DISCUSSÃO

O estudo revelou achados relevantes sobre a inserção do DIU de cobre no pós-parto imediato. A maioria das inserções ocorreu após parto vaginal e foi realizada por médicos, o que pode estar relacionado à maior proporção de partos vaginais nos meses de coleta de dados, à preferência e maior segurança dos profissionais para o procedimento neste contexto e à possíveis intercorrências pós-cesárea que inviabilizam a inserção. A participação limitada de enfermeiras e obstetrizes no processo pode refletir barreiras organizacionais, como a exclusividade médica para a inserção do DIU e a falta de capacitação de outros profissionais, restringindo o potencial da equipe multiprofissional no planejamento reprodutivo^(18,19)^.

Outro achado crítico foi a deficiência na transmissão de informações sobre o DIU às mulheres. Aproximadamente um terço das participantes não recebeu orientações suficientes sobre as vantagens, desvantagens e efeitos colaterais do método, o que pode ter impactado diretamente na continuidade do seu uso e influenciado a preferência por outros contraceptivos. Observou-se que, para muitas mulheres, o primeiro contato com informações sobre o DIU ocorreu apenas durante o trabalho de parto ou parto - momentos potencialmente inapropriados para decisões reprodutivas informadas. Nesse sentido, é fundamental destacar que o trabalho de parto e o parto não são considerados os momentos mais apropriados para a oferta do DIU, uma vez que estes são eventos potencialmente desorganizadores para as parturientes, que se encontram em estado de vulnerabilidade emocional e física e, portanto, a autonomia reprodutiva pode estar sujeita a diversos mecanismos de coerção contraceptiva^(20)^.

Quanto à percepção das usuárias sobre a inserção do dispositivo, pouco mais de um quarto das mulheres relataram dor moderada, dado semelhante ao observado em outro estudo conduzido com adolescentes brasileiras no pós-parto^(21)^. No entanto, não é possível determinar se a dor referida decorre da inserção ou da involução uterina.

Um aspecto que merece atenção é a satisfação com o DIU: 31,2% das mulheres relataram estar satisfeitas e 43,8% muito satisfeitas, o que contrasta com as limitações no aconselhamento e a ausência de oportunidade para escolha de outros métodos contraceptivos. Muitas participantes expressaram preferência por alternativas, como laqueadura ou contraceptivos injetáveis, em momentos distintos. A escolha inicial pela laqueadura pode estar associada à experiência recente do parto e ao desejo de encerrar a fase reprodutiva após o parto, enquanto a posterior preferência por métodos de curto prazo, como o injetável, sugere uma busca por maior controle e flexibilidade. Esses achados, somados ao fato de que uma parcela significativa das mulheres não recebeu orientações adequadas sobre o DIU, e que mais da metade apresentou efeitos colaterais, reforçam a necessidade de fortalecer o acesso à informações e ao aconselhamento no pós-parto. Estratégias mais qualificadas podem promover confiança, motivação e satisfação das usuárias, favorecendo a continuidade no uso do método^(22,23)^. Nesta perspectiva, embora iniciativas governamentais promovam o DIU de cobre como um método altamente eficaz no pós-parto e pós-abortamento, a oferta restrita a um único método e a persuasão para aceitação - muitas vezes vinculadas ao cumprimento de metas por profissionais de saúde - pode gerar um incentivo maior e focado em apenas um método (no caso, o DIU), restringindo o poder de escolha e limitando a autonomia das mulheres, configurando práticas de coerção contraceptiva^(24,25)^.

Além disso, é importante problematizar o não desejo pelo DIU, uma vez que a preferência pela laqueadura no contexto brasileiro reflete tanto construções sociais sobre maternidade quanto experiências negativas com métodos reversíveis, desinformação, receios sobre efeitos colaterais e ausência de diálogo qualificado com profissionais de saúde. Estudos qualitativos poderiam aprofundar a compreensão dos fatores subjetivos e contextuais que influenciam essas escolhas.

No âmbito da atenção à contracepção, a inclusão do parceiro no processo decisório ainda representa um desafio, como evidenciado neste estudo. Para muitas mulheres, a prática contraceptiva é percebida como uma responsabilidade exclusiva, sem divisão com o parceiro, o que reflete desigualdades de gênero historicamente construídas no campo da reprodução^(26-28)^. Estudos qualitativos em profundidade poderiam contribuir para uma melhor compreensão desse processo decisório, explorando não apenas as razões para a ausência da participação da parceria, mas também os fatores que levam algumas mulheres a preferirem decidir sozinhas sobre o uso de métodos contraceptivos.

No que concerne o acompanhamento aos seis meses, a taxa de expulsão do DIU foi de 9,4%, inferior a outras pesquisas nacionais^(21,29)^, enquanto a taxa de remoção foi mais alta do que a relatada em estudos anteriores^(18,30)^. Tais variações podem ser atribuídas a uma série de fatores, incluindo diferenças no perfil populacional, tamanho amostral, técnicas de inserção e estratégias de acompanhamento. A continuidade do uso do DIU ao final do estudo também foi menor do que em outras investigações^(18,29)^, sugerindo que algumas mulheres podem ter sido coagidas a utilizá-lo, como discutido anteriormente.

Um dado particularmente significativo foi o uso excessivo de ultrassonografias transvaginais para verificação do posicionamento do DIU, prática não preconizada pelas diretrizes do Ministério da Saúde^(31)^. Por fim, a restrição da presença de acompanhantes no pós-parto durante a pandemia evidenciou vulnerabilidades adicionais em relação aos direitos sexuais e reprodutivos e ao acesso a serviços de planejamento reprodutivo.

Esses achados devem ser interpretados considerando o modelo assistencial vigente no serviço de saúde investigado, que ainda prioriza intervenções centradas no parto em detrimento de ações contínuas de planejamento reprodutivo. A fragilidade das estratégias de aconselhamento, a carência de protocolos que articulem o cuidado no pós-parto imediato e a limitada atuação multiprofissional podem ter influenciado tanto a adesão inicial ao DIU quanto a sua descontinuidade. Fortalecer o papel educador e promotor da saúde da Enfermagem e de outros profissionais é essencial para garantir um cuidado centrado na mulher, com informações completas e livres de vieses.

### Limitações do estudo

Este estudo apresenta limitações, como a amostra restrita a um único local de investigação e o número reduzido de participantes, reflexo das dificuldades na coleta de dados durante a pandemia de COVID-19. A escassez de insumos e a redução de profissionais qualificados para a inserção do DIU durante esse período podem ter restringido o acesso ao método a um subgrupo específico de mulheres, potencialmente introduzindo viés de seleção e impactando a generalização dos resultados. Ainda assim, a população analisada abrangeu todas as puérperas que inseriram o DIU no hospital, conferindo relevância aos resultados.

Outra limitação relevante do estudo refere-se à elevada perda de seguimento em seis meses (72%), com apenas 32 mulheres completando a segunda etapa. Apesar de terem sido empregadas estratégias para minimizar essas perdas, como múltiplos canais de contato e lembretes periódicos, elas podem ter influenciado a análise dos desfechos. Existe a possibilidade de que parte das perdas esteja associada à descontinuidade do uso ou à insatisfação com o método, o que poderia introduzir viés e superestimar a taxa de continuidade. Embora os dados sugiram que as perdas foram uniformes em relação às características sociodemográficas avaliadas, não se pode excluir a influência de fatores não mensurados. Além disso, o uso de uma amostra não probabilística limita a generalização dos resultados. Por fim, o caráter descritivo da análise não permite inferir associações estatisticamente significativas entre as variáveis avaliadas. Apesar das limitações, o estudo é inovador e evidencia lacunas importantes, como a insuficiência de informações e os aconselhamentos enviesados sobre o DIU no pós-parto imediato por parte dos profissionais de saúde.

### Contribuições para a área da Enfermagem, Saúde ou Política Pública

Os resultados deste estudo podem subsidiar a elaboração e implementação de políticas públicas voltadas à saúde sexual e reprodutiva no pós-parto, com ênfase na oferta de informações adequadas. A disponibilização gratuita de métodos pelo SUS, sem o devido aconselhamento, limita o acesso pleno aos direitos reprodutivos. Além disso, a ínfima participação de profissionais de Enfermagem no cenário estudado evidencia a necessidade de revisitar suas práticas no âmbito do planejamento reprodutivo. É fundamental fortalecer o papel da Enfermagem como educadora e promotora da saúde, com abordagem centrada na mulher e no casal, garantindo orientação qualificada e apoio à tomada de decisão informada.

## CONCLUSÕES

O estudo evidenciou fragilidades nas estratégias institucionais de planejamento e aconselhamento reprodutivo no pós-parto, com aproximadamente metade das mulheres relatando que optaria por outro método em vez do DIU. Muitas não receberam informações suficientes sobre os efeitos colaterais, benefícios, desvantagens e outras opções contraceptivas disponíveis. Notavelmente, para mais da metade das mulheres, o primeiro contato com informações sobre o DIU ocorreu apenas durante o trabalho de parto ou parto, momentos em que a autonomia reprodutiva pode estar comprometida. Fica evidente, portanto, a necessidade de políticas e práticas que ampliem e qualifiquem o aconselhamento contraceptivo no período pós-parto, promovendo escolhas informadas e respeitando as especificidades de cada mulher, com vistas à redução de iniquidades em saúde e desigualdades sociais.

## Data Availability

Os dados de pesquisa só estão disponíveis mediante solicitação.
